# Diagnostic value of progesterone receptor and p53 expression in uterine smooth muscle tumors

**DOI:** 10.1186/1746-1596-7-1

**Published:** 2012-01-05

**Authors:** Iman H Hewedi, Nehal A Radwan, Lobna S Shash

**Affiliations:** 1 Pathology Department, Faculty of Medicine, Ain Shams University, Cairo, Egypt

**Keywords:** PR, p53, uterine smooth muscle tumor, Leiomyosarcoma, STUMP

## Abstract

**Background:**

The diagnosis of uterine smooth muscle tumors depends on a combination of microscopic features. However, a small number of these tumors still pose difficult diagnostic challenges.

**Aim:**

To investigate progesterone receptor (PR) and p53 expression in leiomyomas (LMs), atypical leiomyomas (ALMs), smooth muscle tumors of uncertain malignant potential (STUMP), and leiomyosarcomas (LMSs) and to evaluate the potential utility of the selected immunohistochemical markers in differentiating these tumors.

**Materials and methods:**

Immunohistochemical expression of PR and p53 was investigated in 41 uterine smooth muscle tumors comprising: 15 LMS, 4 STUMP, 6 ALM and 16 LM. Quantitative evaluation of PR and p53 expression was graded on a scale from 0 to 3+.

**Results:**

Leiomyosarcomas showed reduced PR expression. All LMs as well as ALMs and STUMP were stained intensely for PR. Conversely, LMS was strongly stained with p53, while the three non-sarcomatous groups (STUMP, ALM, LM) were either entirely negative or weakly stained for p53. Regarding both PR and p53 expression, the difference between the LMS group and the three non-sarcomatous groups was highly significant (p < 0.001). Combined high PR - low p53 expression was seen in all the 26 examined cases of the non-sarcomatous group including the STUMP cases and none of the LMS cases. Therefore, it represents a "benign" profile with 100% specificity in diagnosis of a non-sarcomatous tumor.

**Conclusion:**

Immunohistochemistry for PR and p53 is valuable as an adjunct tool to morphological assessment of problematic uterine smooth muscle tumors.

## Introduction

Uterine smooth muscle tumors are the most common female genital tract neoplasms. They are classified into: leiomyomas (LMs), smooth muscle tumors of uncertain malignant potential (STUMP) and leiomyosarcomas (LMS) [1]. LM is the most common type which occurs in nearly 40% of women older than 35 years. Uterine LMSs are relatively rare smooth muscle tumors, accounting to approximately one third of uterine sarcomas and 1.3% of all uterine malignancies [2].

Most of the uterine smooth muscle tumors are readily classified into benign or malignant, using a combination of microscopic features including the presence and type of necrosis, the degree of cytologic atypia, the mitotic activity, and the relationship of the tumor to surrounding normal structures. However, a small number of uterine smooth muscle tumors constitute difficult diagnostic challenges. Morphologically, some variants of LM, such as cellular leiomyoma, atypical leiomyoma (leiomyoma with bizarre nuclei) (ALM) and mitotically active leiomyoma can mimic malignancy in one or more aspects. Also, some smooth muscle tumors that cannot be classified as benign or malignant based on histopathological criteria are diagnosed as the smooth muscle tumor of uncertain malignant potential (STUMP). This term is used when there is some significant doubt about the failure probability associated with a particular combination of microscopic features. The ultimate biological behavior of tumors classified as STUMP remains uncertain. Thus, it would be clinically valuable to decrease the percentage of these cases for the optimal management of the patients [2,3].

Variations in interpretation and subjective identifications of some microscopic features (mitotic figures, type of necrosis) have resulted in introducing potential diverse diagnostic criteria for uterine smooth muscle tumors. Few reports investigated steroid receptor expression in uterine smooth muscle tumors. Significant differences of PR expression between uterine LM, STUMP and LMS were observed [4-8]. Therefore, We explored the PR expression in LMs, ALMs, STUMP, and LMSs to determine whether PR might be of diagnostic value in the assessment of problematic uterine smooth muscle tumors or not. We also stained the cases with p53 to investigate whether it might be of complementary value to PR.

## Materials and methods

### • Tissue collection

This retrospective study included 41 uterine smooth muscle tumors. Cases were retrieved from the archives of the Early Cancer Detection Unit, Ain Shams Obstetrics and Gynaecology Hospital, Cairo, Egypt. All the specimens had been routinely fixed in formalin and processed in paraffin wax. The cases were reviewed and the histological diagnosis was assigned according to the recently published criteria [1]. After ruling out infarcted and mitotically active leiomyomas, the histopathologic diagnostic criteria applied in this study are summarized in Table 1. All atypical leiomyoma, STUMP and leiomyosarcoma cases received between 2005 and 2010 were included, while a comparable number of LMs received during the same period, were randomly sampled. Accordingly, cases included 16 leiomyomas, 6 atypical leiomyomas, 4 STUMP and 15 leiomyosarcomas. The study was carried out with full local ethics approval.

**Table 1:** Histologic criteria employed in this study for the diagnosis of uterine smooth muscle tumors

Diagnosis	Tumor cell necrosis	Atypia	MF/10 HPF*
Leiomyosarcoma	Present	Diffuse moderate to severe	Any level
	
	Present	None to mild	≥ 10
	
	Absent	Diffuse moderate to severe	≥ 10

STUMP	Present	None to mild	< 10
	
	Absent	Diffuse moderate to severe	5-9 or atypical mitotic figures
	
	Absent	Focal moderate to severe	≥ 5

Atypical leiomyoma	Absent	Focal or diffuse moderate to severe	< 5

Leiomyoma	Absent	None to mild	< 5

*Mitotic figures per 10 High power field

### • Immunohistochemistry

Immunohistochemical analysis for PR and p53 with a labelled streptavidin- biotin-peroxidase complex technique was performed on formalin-fixed and paraffin-embedded tumor sections. Commercially available ready to use rabbit monoclonal antibody against PR (Cell Marque- CA- USA- Cat. #323R-18) and mouse monoclonal antibody against p53 (Lab vision- CA- USA- Cat. # MS-104-R7) were used in this study. Antigens were retrieved by microwaving in citrate buffer for 20 minutes for PR and p53. The final reaction product was developed with diaminobenzidine. Proper positive and negative controls were performed.

### • Immunohistochemical Analysis

The immunohistochemical preparations were assessed by the three authors using a multi-headed microscope. Only nuclear staining was considered as a positive reaction for PR and p53. Due to different staining properties, the assessment of the degree of immunohistochemical staining was made according to two scoring scales based on the percentage of the stained cells as described by Gökaslan et al. [5]. The quantitative evaluation of PR was made as follows: 3+ for > 50% of the cells immunostained, 2+ between 10 and 50%, 1+ for < 10% and 0 (none) for no staining. At the same time, p53 was evaluated as: 3+ for > 20% of the cells immunostained, 2+ between 5 and 20%, 1+ for < 5% and 0 (none) for no staining. For both immunostains, 0 and 1+ are regarded as low expression, while 2+ and 3+ are regarded as high expression.

### • Statistical analysis

Chi-square tests were used to compare the frequency distributions of PR and p53 expression between the analyzed tumor groups. *P *values of less than 0.05 were considered statistically significant and those less than 0.01 were highly significant. IBM SPSS statistics (V. 19.0, IBM Corp., USA, 2010) was used for data analysis.

## Results

**Figure 1:**
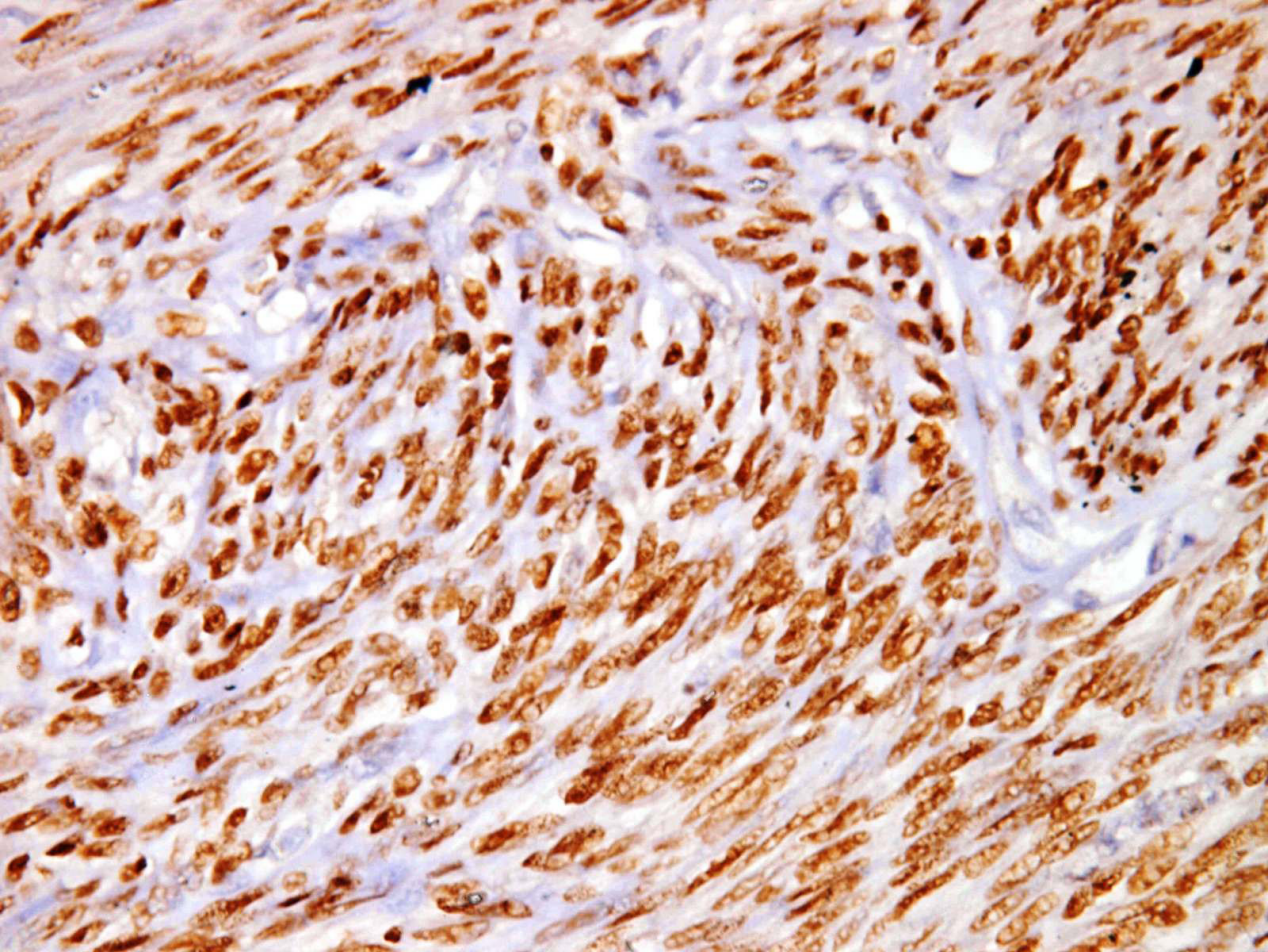
**High PR expression (3+) in a leiomyoma,(PR × 400)**.

**Figure 2:**
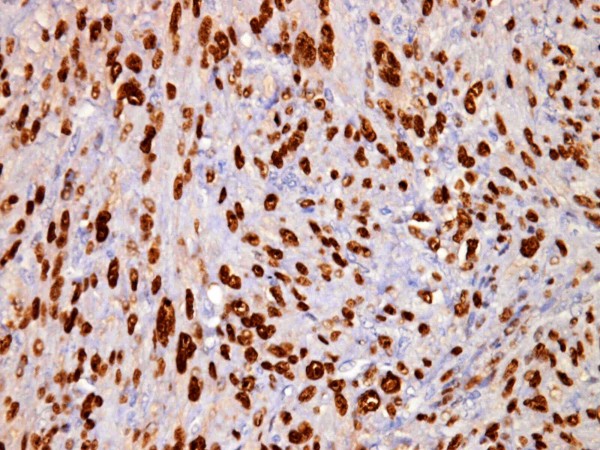
**Atypical leiomyoma with high PR expression (3+),(PR × 400)**.

**Figure 3:**
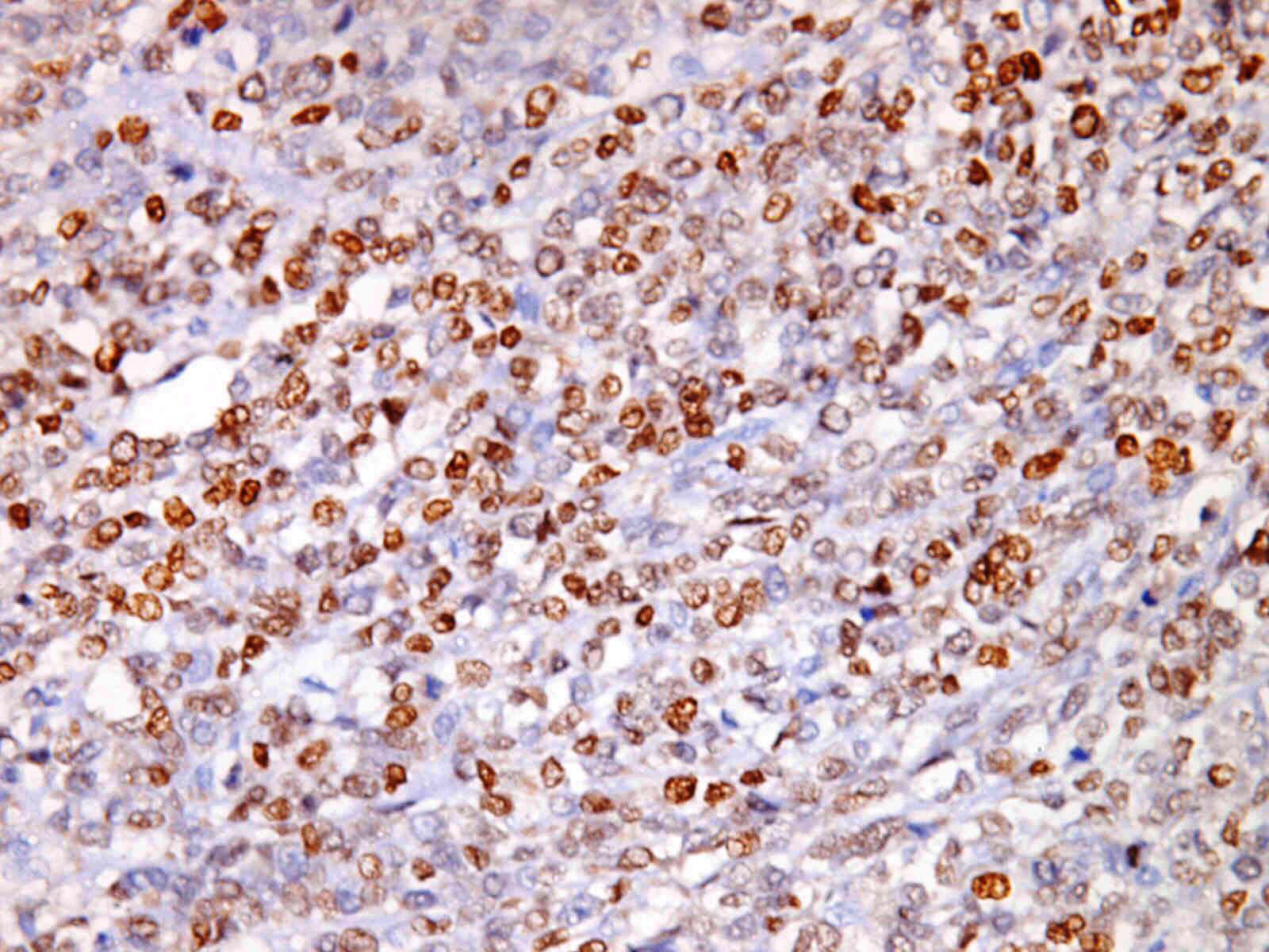
**Smooth muscle tumor of uncertain malignant potential (STUMP) with high PR expression (3+),(PR × 400)**.

**Figure 4:**
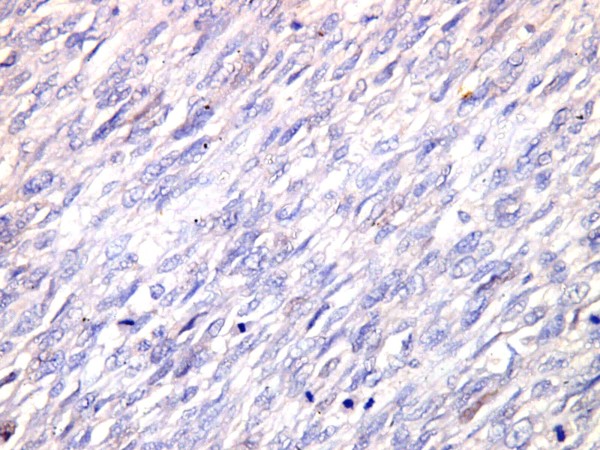
**A leiomyosarcoma exhibits negative immunoreactivity for PR,(PR × 400)**.

**Figure 5:**
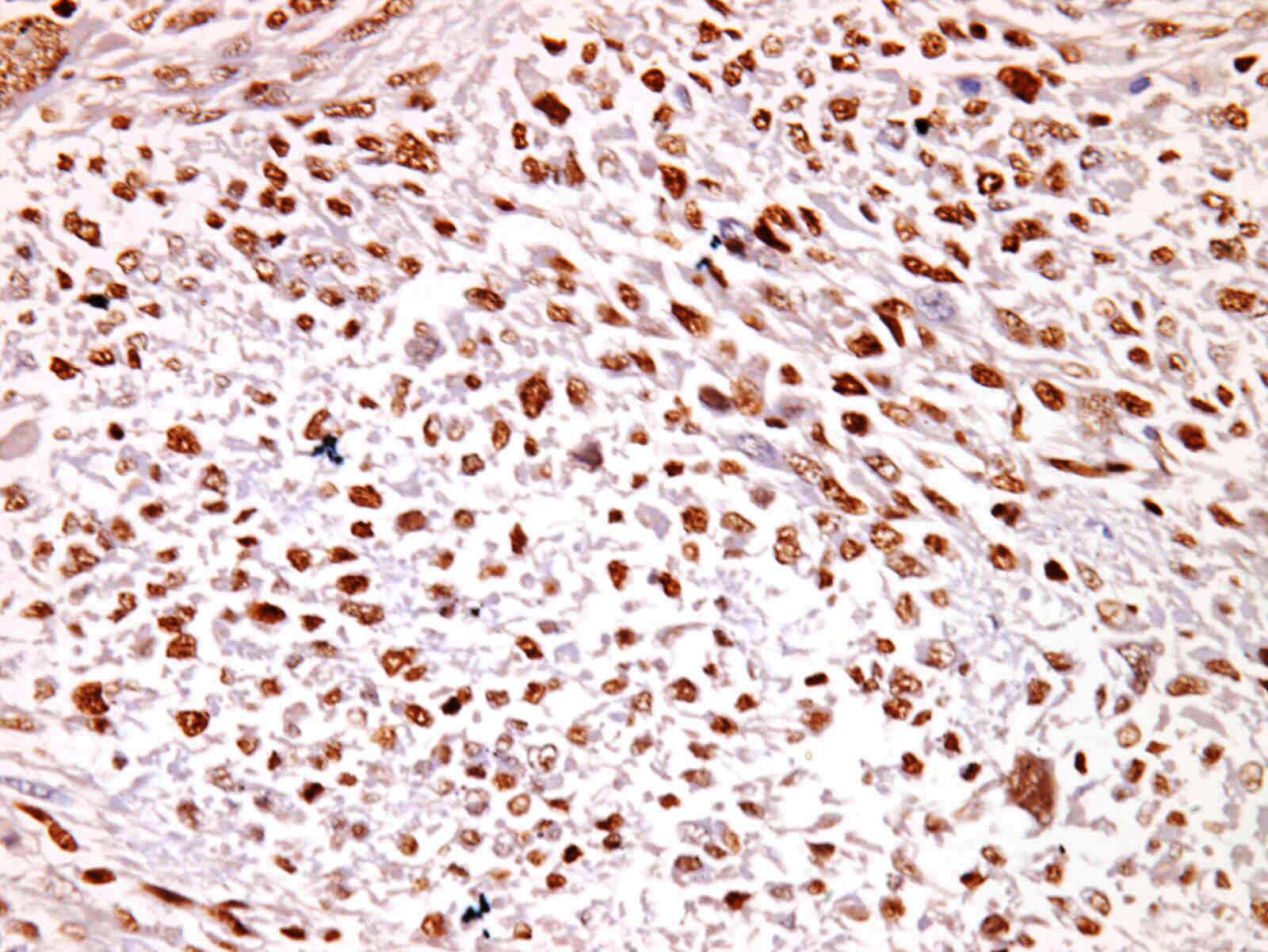
**High p53 expression (3+) in a leiomyosarcoma,(p53 × 400)**.

Results are shown in Tables 2 and 3 in addition to Figures 1, 2, 3, 4, and 5.

**Table 2:** Expression of PR and p53 in uterine smooth muscle tumors

Tumor type	PR expression				P53 expression			
	
	*High -expression*		*Low-expression*		*High-expression*		*Low-expression*	
	
	+3	+2	+1	0	+3	+2	+1	0
***Non-sarcomatous tumors:***	***24/26***	***2/26***	***0/26***	***0/26***	***0/26***	***0/26***	***10/26***	***16/26***
• Leiomyoma	16/16	0/16	0/16	0/16	0/16	0/16	4/16	12/16
• Atypical leiomyoma	5/6	1/6	0/6	0/6	0/6	0/6	2/6	4/6
• STUMP*	3/4	1/4	0/4	0/4	0/4	0/4	4/4	0/4

***Leiomyosarcoma ***	***0/15***	***1/15***	***1/15***	***13/15***	***7/15***	***8/15***	***0/15***	***0/15***

*STUMP = smooth muscle tumor of unknown malignant potential

**Table 3:** combined PR-p53 expression profile in uterine smooth muscle tumors

Tumor type	Combined PR- p53 expression profile		
	
	High PR- Low p53	Low PR- High p53	High PR- High p53
Non-sarcomatous tumors (LM+ALM+STUMP)	26/26	0/26	0/26

LMS	0/15	14/15	1/15

*Abbreviations: LM = leiomyoma, ALM = atypical leiomyoma, STUMP = smooth muscle tumor of unknown malignant potential, LMS = Leiomyosarcoma

X^2^= 41.00, p < 0.001

Leiomyosarcoma showed reduced PR expression. Negative PR expression was noted in 13 out of 15 LMSs. None of the LMSs studied exhibited 3 + PR positivity. However, all LMs showed intense staining for PR (all samples, 3+). The ALMs and STUMP were also intensely stained for PR (all samples 2+ or 3+). The difference between the LMS group on one hand and the combined three non-sarcomatous groups (LM, ALM, and STUMP) on the other hand, regarding PR expression this difference proved to be highly significant (x^2 ^= 38.126, p < 0.001). The difference of PR expression among LMSs in comparison to each individual, non- sarcomatous group was also highly significant with p < 0.001 (LMS vs. STUPM; x^2 ^= 15.99, LMS vs. ALM; x^2 ^= 18.55, LMS vs. LM; x^2 ^= 31.00).

On the contrary, the malignant side of the spectrum (LMS) was strongly stained with p53 (all samples 2+ or 3+), while the three non-sarcomatous groups were either entirely negative or weakly stained for p53.

The difference between the LMS group on one hand and the combined three non -sarcomatous groups (LM, ALM, and STUMP) on the other hand, regarding p53 expression was also highly significant (x^2 ^= 41.00, p < 0.001). This highly significant difference, regarding p 53 expression (p < 0.001) was also extended on comparing LMS to each of the non-sarcomatous groups (STUMP, ALM, and LM) individually with x^2^= 19.00, 21.00 and 31.00 respectively.

On exploring the combined results of the used markers, it was evident that the profile of high PR expression (2+ or 3+) and low p53 expression (0 or 1+) is 100% specific for the non-sarcomatous diagnosis. This "benign" profile of high PR- low p53 expression is seen in all the 26 examined non sarcomatous cases including the STUMP and in none of the LMS cases. The single LMS case that showed high PR expression (2+) also exhibited intense p53 staining (3+).

## Discussion

Previous studies have shown that the distinction between LMS, STUMP and ALM may sometimes be problematic because of inaccurate and inconsistent definitions in diagnostic criteria [9,10]. Earlier reports have investigated PR and p53 expression in uterine smooth muscle tumors [5-7,11,12]. Yet, the current study is the leading one to study these two markers among diverse uterine smooth muscle tumors subsequent to application of the recent diagnostic criteria published in 2011 by Zaloudek et al [1]. Additionally, we chose to apply a simple, yet efficient method to assess the immunostaining results that can be used not only in academic settings, but also in clinical practice.

PR is routinely determined in patients with breast and endometrial cancer [6]. Previous reports had shown that LMs are hormone- dependent tumors and for the last few years investigators focused on LMS, STUMP and specific subtypes of LMs [6-8,13-16]. Little is still known about the progesterone receptor expression pattern of ALMs, and STUMP [5]. Gökaslan et al. stated that all their nine ALMs were immunopositive for PR with a moderate-to-strong staining intensity [5]. Zhai and colleagues observed a strong positive staining for PR in all of the eight STUMP cases included in their study [8]. Likewise, Mittal and Demopoulos detected immunopositivity for PR in all of their seven STUMP cases [7]. Bodner et al. detected PR expression in 17 of their 24 STUMP cases [6]. We found PR expression in all of our 16 LM cases, and in our series ALMs and STUMP showed a markedly similar PR staining pattern to that of LMs.

Most researchers detected low immunostaining rates of PR in LMSs [6,11,17]. We have detected a prominent reduction in PR expression in LMS cases where only a couple of cases showed progesterone receptor expression with slight-to-moderate intensity. Bodner et al. [15] reported that progesterone receptors were expressed in 43% of the leiomyosarcoma cases; a percentage which is much higher than the one detected in this study. However, they also reported that such expression did not influence the prognosis of LMS. The use of earlier diagnostic criteria in Bodner et al. study might allow inclusion of few STUMP cases among their leiomyosarcomas. This could be explained in view of the fact that previous diagnostic criteria for smooth muscle tumors [18] did not allow the presence of tumor cell necrosis into the STUMP category and pushed them into leiomyosarcomas which has been modified into the recent criteria applied in the current study [1].

The present study found a prominent difference in PR expression between LMS and STUMP. Meanwhile, the staining pattern of the STUMP group was strikingly similar to that of LM. These results were in concordance with the results of Mittal & Demopolous [7] and Petrovic et al. [12]. In addition, our study also demonstrated that ALMs had a similar intensive and strong staining pattern of progesterone receptors to that of LM. We suggest that progesterone receptor expression analysis can aid us in effectively distinguishing both ALMs and STUMP from LMSs.

This study also investigated the expression of p53; a suppressor gene that was very commonly found in leiomyosarcomas. All our Leiomyosarcoma samples were almost equally divided into either 2+ or 3+ regarding p53 immunostaining. Mittal and Demopoulos [7] reported 5 out of 12 LMS cases which expressed p53 in ≥ 15% of the cells while 4 LMS cases showed positivity in ≥ 40% of the cells in their study.

In the female genital tract and breast tumors, Westhof et al. [19] assumed that elevated levels of p53 protein might indicate p53 gene mutations. They concluded that p53 expression and overexpression were organ-dependent. De Vos et al. [20] were the first to suggest that p53 mutations are more frequent in leiomyosarcomas. They claimed that the acquisition of p53 mutation was the one distinguishing difference between leiomyomas and leiomyosarcoma. Hong et al. [21] stated that even though p53 expression in leiomyosarcoma was significantly higher than leiomyoma, the frequency of p53 positivity was not as high as expected. Jeffers et al. [22] claimed that positive immunohistochemistry did not always correlate positively with the presence of mutation. Nordal et al. [23] indicated that p53 alterations might play an important role in the carcinogenesis of uterine sarcomas. Nonetheless, they also emphasized that p53 accumulation had no impact on prognosis. Wang et al. [24] proposed p53 could be applied as an accessory criterion in the differential diagnosis of smooth muscle tumors of the uterus. Kayser et al. [25] claimed that benign metastasizing leiomyoma showed p53 over expression.

We observed intense staining of our leiomyosarcoma samples and poor staining of all the rest of the smooth muscle tumors, including STUMP cases. These findings were primarily in accordance with the study of Mittal and Demopoulos [7]. According to our study, p53 was more clearly shown to be an indicator of malignancy with strong statistical significance.

In keeping with the excellent prognosis in patients with STUMP previously reported [1,7], the immunoprofile of STUMP for PR and p53 in this series was much closer to leiomyomas than leiomyosarcomas. It would be of interest to know if the immunoprofile of an occasional STUMP behaving in a malignant fashion is closer to that of leiomyosarcomas than to that of leiomyoma.

The findings of the current and previous studies can be used to evaluate cases of uterine smooth muscle tumors in which histologic findings are ambiguous or borderline. These would include tumors where mitotic figures are clumped or poorly formed, and thus difficult to identify. Smooth muscle tumors, in which nuclear atypia is moderate to severe with 5 to 9 mitoses/10 HPF can also be further evaluated. Moreover, they can be used to evaluate cases in which only a small sample of the tumor is available for study. As to the evaluation of any lesion, the immunohistochemical findings of combined PR and p53 expression should be used in concert with clinical, gross, and light microscopic findings to arrive at a final diagnosis.

Although leiomyosarcomas are usually negative for PR, occasional cases showed positive staining for these receptors. Hormonal management in these cases might be helpful in controlling these tumors.

In conclusion, PR and p53 immunostaining profile is useful in distinguishing leiomyosarcomas from STUMP and atypical leiomyomas. So we can improve the objectivity and raise the degree of certainty concerning the histopathologic decision; allowing for optimal management of such tumors.

## Competing interests

The authors declare that they have no competing interests.

## Authors' contributions

IHH conceived, designed and coordinated the study, evaluated immunohistochemistry, performed the statistical analysis and drafted the manuscript. NAR reviewed the histological diagnosis, evaluated immunohistochemistry, carried out photographing, participated in the study design and helped to draft the manuscript. LSS participated in the sequence alignment, performed data collection, evaluated immunohistochemistry and critically reviewed the manuscript. All authors read and approved the final manuscript.
